# The BaeSR two-component system activates *bamK*, a paralog of the essential β-barrel foldase *bamA*, in *Klebsiella pneumoniae*

**DOI:** 10.1128/mbio.03497-24

**Published:** 2026-03-23

**Authors:** Kelly M. Storek, Janina Reeder, Dawei Sun, Donghong Yan, Austin K. Murchison, Min Xu, Elizabeth Skippington, Steven T. Rutherford

**Affiliations:** 1 Department of Infectious Diseases, Genentech, Inc. 162120 , South San Francisco, California, USA; 2 Department of Bioinformatics, Genentech, Inc. 162120 , South San Francisco, California, USA; 3 Department of Structural Biology, Genentech, Inc. 162120 , South San Francisco, California, USA; 4 Department of Translational Immunology, Genentech, Inc. 162120 , South San Francisco, California, USA; Monash University, Melbourne, Australia

**Keywords:** *Klebsiella*, BamA, BamK, outer membrane proteins, outer membrane, BaeSR

## Abstract

β-Barrel outer membrane proteins (OMPs) perform critical functions in gram-negative bacteria including outer membrane biogenesis, nutrient acquisition, and efflux. OMPs are folded by the β-barrel assembly machinery (BAM) with BamA as a central and essential component. BamK, a *Klebsiella pneumoniae* BamA paralog, can functionally replace BamA; however, native *bamK* expression and a role for BamK have not been reported. Here, we identify mutations in a two-component signal transduction system that activate expression of *bamK* in *K. pneumoniae*. Selections for *K. pneumoniae* mutants resistant to a BamA-targeting antibacterial macrocyclic peptide, PTB1-1, yield on-target and off-target mutations. On-target *bamA* mutations overlap with previously described positions in the PTB1-1 binding site. Off-target mutations map to three distinct genes, *oqxR*, *ramR*, and *baeS*. Transcriptomics analyses indicate that mutations in *baeS,* a sensor histidine kinase, activate *bamK* expression, and these mutants render *bamA* dispensable for bacterial growth. Our results demonstrate that BamK can functionally substitute for the essential function of BamA *in vitro* and *in vivo,* and *bamK* expression is controlled by a two-component signal transduction system that responds to envelope stress in gram-negative bacteria. These findings provide insight into possible roles for BamK and considerations for future efforts to develop antibiotics targeting OMP folding.

**IMPORTANCE:**

The complex envelope of gram-negative bacteria is a critical structure. It is assembled and maintained by multiple essential pathways, all of which, including the β-barrel assembly machinery (BAM) complex, have been the focus of novel antibiotic discovery efforts. Species in the genus *Klebsiella* encode a paralog to the BAM complex component BamA, called BamK, but the importance and role of this protein has remained a mystery. Leveraging mutants resistant to a recently discovered BamA inhibitor, we describe how activation of the BaeSR envelope stress response system can activate *bamK* expression to overcome the loss of BamA or its function both *in vitro* and *in vivo*. These findings provide important insights into BamK, *Klebsiella* biology, gram-negative stress responses, and targeting outer membrane protein folding as an antibacterial strategy.

## INTRODUCTION

*Klebsiella pneumoniae*, a ubiquitous gram-negative bacterium, causes deadly infections in humans (1). Treatment options for these infections have been threatened by the spread of antibiotic resistance, which has resulted in strains of *K. pneumoniae* impervious to all available drugs (2). Antibacterial compounds with novel targets or unique mechanisms of action are needed to overcome this threat to public health.

A hurdle to the discovery of antibiotics active against *K. pneumoniae* and other gram-negative bacteria is the outer membrane. Unlike typical biological membranes, the outer membrane is asymmetrical with phospholipids in the inner leaflet and lipopolysaccharide (LPS) in the outer leaflet (3). Lateral interactions between adjacent LPS molecules coordinated by divalent cations and hydrophobic packing of the LPS acyl chains establish a distinct permeability barrier that prevents access to toxigenic molecules, providing intrinsic resistance to many antibiotic classes (4, 5). Targeting essential processes in the outer membrane of gram-negative bacteria has been pursued as a strategy to circumvent this barrier (6–12).

β-Barrel outer membrane proteins (OMPs) carry out critical cellular functions, including importing essential metabolites and nutrients, effluxing toxic molecules, transporting LPS, constructing the outer membrane, transporting lipoprotein, and responding to stress (4, 13). The multiprotein β-barrel assembly machine (BAM) is responsible for folding and inserting OMPs into the outer membrane (14–16). BamA, the central component of the BAM complex and itself an OMP, is essential for viability and conserved across gram-negative bacteria (16). OMPs are translated in the cytoplasm, secreted through the Sec apparatus, and chaperoned across the periplasm before interacting with BamA and the BAM lipoproteins, BamBCDE, for folding and insertion in a process that occurs in the absence of an obvious energy source (4). Solved structures, molecular dynamic simulations, and biochemical and genetic analyses indicate that the lateral gate seam formed between the first and last β-strands of the BamA β-barrel is critical for its OMP folding activity (13, 17–22). Efforts to find inhibitors of BamA activity have identified the inhibitory antibody MAB1, the lectin-like bacteriocin LlpA, the cationic small molecule amphiphile MRL-494, the natural products darobactin and dynobactin, and the macrocyclic peptides PTB1-1, PTB2-2, CP1, CP2, and CP3 (6, 8, 9, 11, 23–27). In addition to validating BamA as a potential antibacterial target, these inhibitors have proven useful as tools to dissect the molecular mechanism of OMP folding by BamA in gram-negative bacteria.

*Klebsiella* species, including *K. pneumoniae*, *K. quasipneumoniae*, *K. varicola*, and some closely related species, encode a BamA paralog, called BamK, in their core genomes (28). BamK has not been described outside this restricted clade and likely originated from a duplication of an ancestral *bamA* gene (28). Despite only 63% amino acid identity with BamA, BamK is functionally equivalent to BamA. It forms a complex with the four lipoprotein components of the BAM complex, folds OMPs with similar kinetics to BamA, and supports the growth of *K. pneumoniae* in the absence of *bamA* (28). Under previously tested laboratory conditions, *bamK* is dispensable and not expressed, leaving its biological relevance an open question (28).

Using the potent and selective BamA-inhibiting macrocyclic peptide PTB1-1 (26), we identify mutants that lead to resistance via increased expression of *bamK* in *K. pneumoniae*. Specifically, mutations in the gene encoding the sensor histidine kinase of the BaeSR two-component signal transduction system increase *bamK* expression >250-fold. Induced expression of *bamK* is sufficient to support growth in the absence of *bamA* and provide complete resistance to PTB1-1. Differences in the extracellular loop sequences of BamA and BamK likely allow *K. pneumoniae* to avoid inhibitory interactions. These results allow for novel insight into the potential ecological role of BamK in an important human pathogen and can guide antibacterial efforts aimed at targeting β-barrel folding.

## RESULTS

### *Klebsiella pneumoniae* has an elevated frequency of resistance to PTB1-1

**Fig 1:**
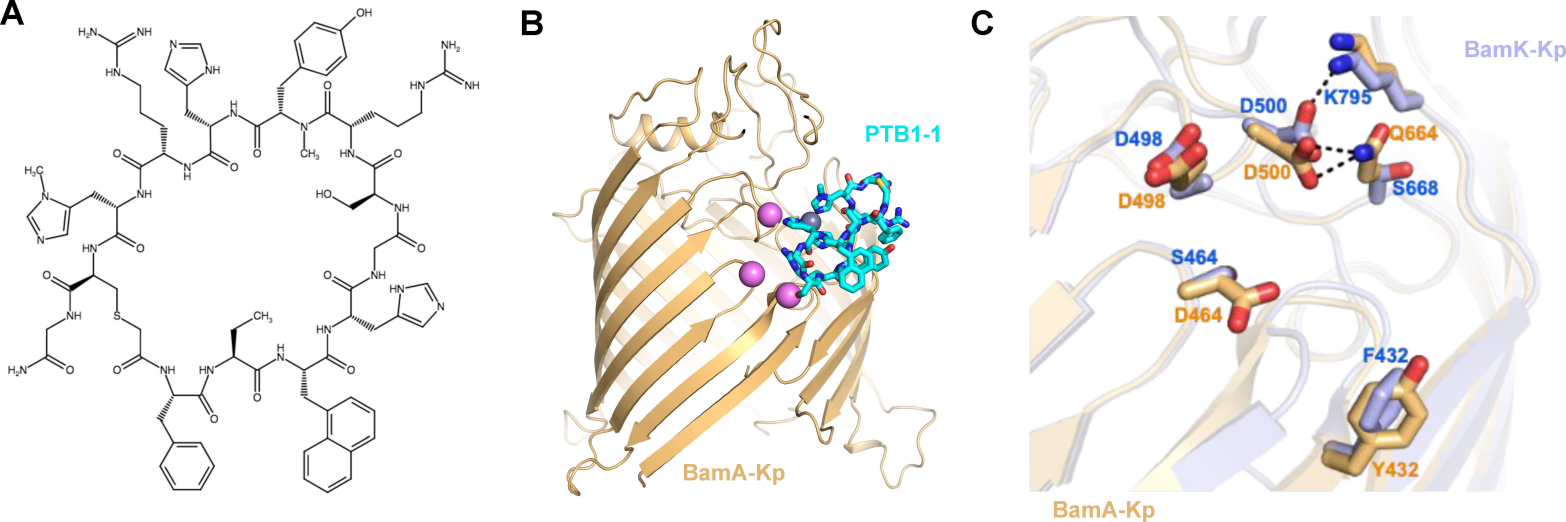
The antibacterial macrocyclic peptide PTB1-1 targets BamA in *K. pneumoniae*. (**A**) Chemical structure of the antibacterial macrocycle PTB1-1 (26). (**B**) Structural model of the PTB1-1 binding site on *K. pneumoniae* BamA (BamA-Kp, AlphaFold model AF-A0A378C0J7-F1) based on resistance mutation mapping (Table 2 and Fig. S1) and the reported structure of PTB1-1 bound to *E. coli* BamA (PDB: 9CNX [26]). BamA is gold, PTB1-1 is cyan, and positions where substitutions in *K. pneumoniae* BamA lead to PTB1-1 resistance are pink spheres. (**C**) Comparison of *K. pneumoniae* BamA (BamA-Kp, gold, AlphaFold model AF-A0A378C0J7-F1) and the homologous region of BamK (BamK-Kp, blue, AlphaFold model [see Materials and Methods]) at the reported *E. coli* BamA PTB1-1 binding site (26).

The macrocyclic peptide PTB1-1 (Fig. 1A) exhibits selective gram-negative antibacterial activity (Table 1) by interfering with the OMP folding activity of BamA (26). PTB-1 is active against closely related gram-negative species, including *E. coli, K. pneumoniae, K. aerogenes*, and *E. cloacae*, but inactive against the more divergent gram-negative pathogens *P. aeruginosa* and *A. baumannii*. Importantly, gram-positive bacteria do not encode *bamA* and, accordingly, *B. subtilis* and *S. aureus* are insensitive to PTB-1.

**TABLE 1:** Minimal inhibitory concentrations (MICs) for the BamA-targeting antibacterial macrocycle PTB1-1 against wild-type bacterial strains

Bacterial species and strain	PTB1-1 MIC (µM)
*E. coli* ATCC 25922	6.25
*E. coli* BW25113	6.25
*Klebsiella pneumoniae* ATCC 700721	12.5
*Klebsiella pneumoniae* ATCC 43816	6.25
*Klebsiella aerogenes* ATCC 13048	12.5
*Enterobacter cloacae* ATCC 222	0.2
*Enterobacter cloacae* ATCC 13047	50
*Pseudomonas aeruginosa* PA14	>100
*Acinetobacter baumannii* ATCC 19606	>100
*Staphylococcus aureus* USA300	>100
*Bacillus subtilis* 168	>100

Spontaneous resistance to PTB1-1 in *Escherichia coli* ATCC 25922 occurred at a frequency of resistance (FOR) of 1.4 × 10^−8^–5.2 × 10^−8^. The *bamA* gene was Sanger sequenced in all spontaneously resistant *E. coli* isolates and confirmed to possess coding mutations in *bamA* (Table 2), confirming previous resistance mutant analyses (26). We expanded the resistance investigation by selecting for *K. pneumoniae* ATCC 43816 and *K. pneumoniae* ATCC 700721 spontaneous resistant mutants able to grow in the presence of PTB1-1. Selections were performed in two different rich media (MHB and LB) and resulted in FORs that were 3- to 35-fold higher for *K. pneumoniae* ATCC 700721 compared to *E. coli* ATCC 25922 (Table S1).

**TABLE 2:** Minimal inhibitory concentrations (MICs) for the BamA-targeting antibacterial macrocycle PTB1-1 against *E. coli* and *K. pneumoniae* isolates with spontaneous resistance-conferring mutations in *bamA*

BamA substitutions in resistant mutants	PTB1-1 MIC (µM)		
	*E. coli* ATCC 25922	*K. pneumoniae* ATCC 700721	*K. pneumoniae* ATCC 43816
WT	6.25	12.5	6.25
G437C	>50	>50	n.d.^*a*^
G437S	>50	>50	n.d.
D500A	>50	>50	n.d.
D500N	>50	n.d.	>50

^*a*^ n.d. (not determined) indicates that an isolate encoding this BamA variant was not selected with this strain background in these experiments.

To identify the causative mutation, the *bamA* gene was amplified from 70 *K. pneumoniae* ATCC 700721 and ATCC 43816 PTB1-1-resistant mutants and Sanger sequenced (Fig. 1B and Fig. S1A). Six PTB1-1-resistant mutants encoded five distinct mutations in *bamA*, each of which was previously isolated at conserved *bamA* positions in *E. coli* PTB1-1 resistance studies (26) (Fig. S1 and Table 2). These mutations led to amino acid alterations in BamA extracellular loops located at or near the lateral gate that define the structurally determined PTB1-1 binding site (26) (Fig. 1B and Fig. S1B).

In contrast to *E. coli* where all identified PTB1-1-resistant mutations were in *bamA*, >90% of the resistant *K. pneumoniae* isolates encoded wild-type *bamA*. Whole genome sequencing was performed to identify the potential mechanisms of resistance for 51 PTB1-1-resistant *K. pneumoniae* mutants that lacked a mutation in *bamA* (i.e., *bamA*^WT^). More than one off-target PTB1-1-resistant mutation was identified in three different genes: *baeS*, *ramR*, and *oqxR* (Tables S2 and S3). *baeS* encodes the sensor histidine kinase of the BaeSR two-component signal transduction system (29–31), *ramR* encodes a TetR-family transcription factor, and *oqxR* encodes a Rrf2-family transcription factor (32, 33). Homologs of *ramR* and *oqxR* are not found in laboratory *E. coli* strains, including the strains used for PTB1-1-resistance studies, *E. coli* ATCC 25922 and *E. coli* BW25113, rationalizing why these mutations were not previously identified. However, the BaeSR two-component system is conserved across the *Enterobacteriaceae* and is important for envelope stress response in both *E. coli* and *K. pneumoniae* (34). Despite strong sequence conservation of *baeS*, 76.8% amino acid identity and 84.3% amino acid similarity between *E. coli* ATCC 25922 and *K. pneumoniae* ATCC 43816, all PTB1-1-resistant *E. coli* mutants possessed coding mutations exclusively in *bamA* (26). Thus, our selections identified additional mechanisms of resistance to BamA inhibition by PTB1-1 that appear to be *K. pneumoniae* specific.

**Fig 2:**
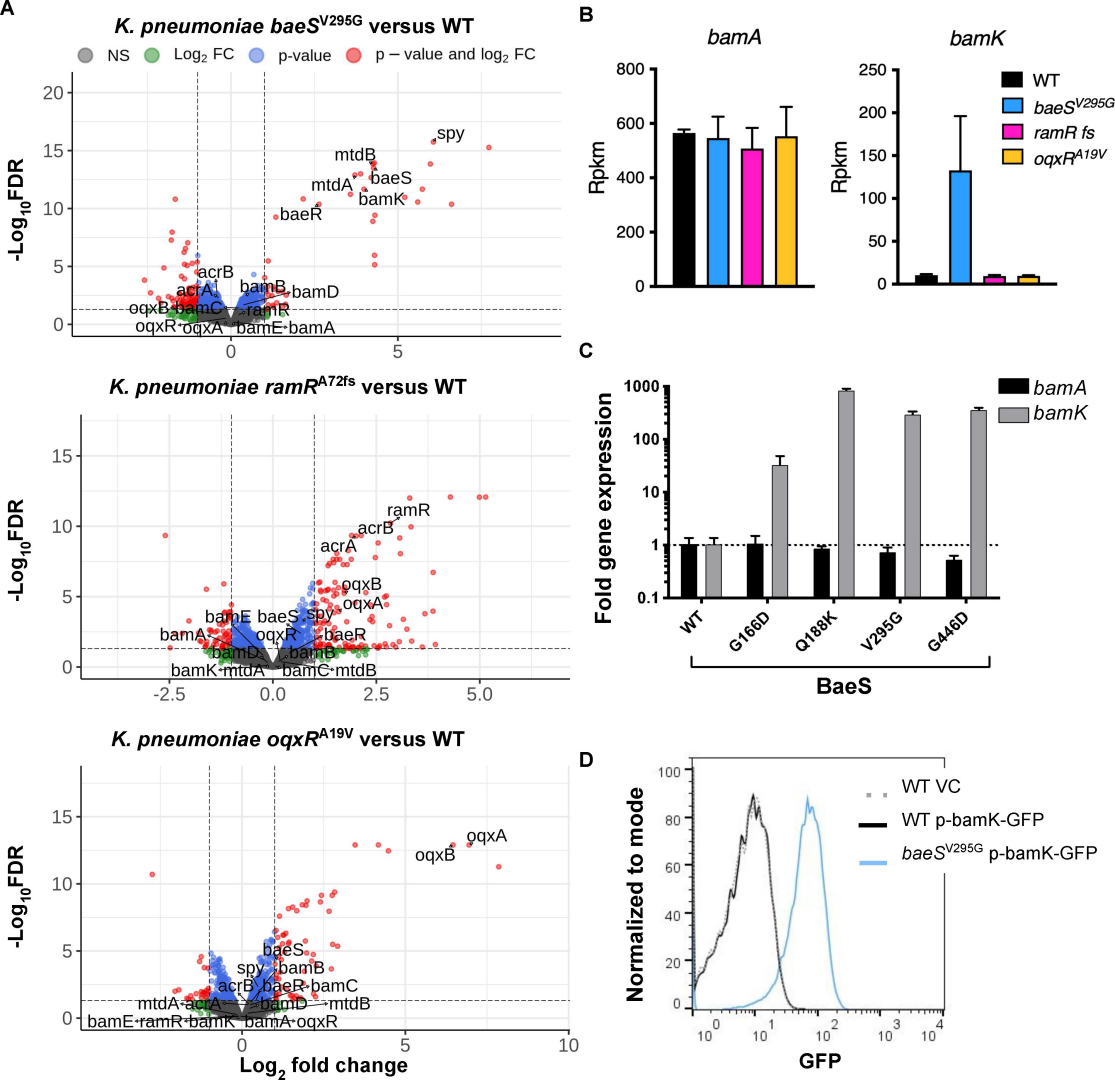
Expression of *bamK* is induced in *baeS* mutants conferring PTB1-1 resistance to *K. pneumoniae* ATCC 43816. (**A**) Volcano plots of differential gene expression profiles from pairwise contrasts of *baeS*^V295G^ vs WT (left), *ramR*^A72fs^ vs WT (middle), and *oqxR*^A19V^ vs WT (right). Red indicates transcripts passing the FDR ≤ 0.05 and |log2 FC| ≥ 1 threshold, blue indicates transcripts passing the FDR ≤ 0.05 threshold, green represents transcripts passing the |log2 FC| ≥ 1 threshold, and gray represents the remaining transcripts. (**B**) Transcript levels (RPKM, reads per kilobase of transcript per million mapped reads by RNAseq) for *bamA* (left) and *bamK* (right) in wild-type *K. pneumoniae* (black) or *K. pneumoniae baeS*^V295G^ (blue), *K. pneumoniae ramR*^A72fs^ (pink), and *K. pneumoniae oqxR*^A19V^ (yellow) spontaneous PTB1-1-resistant mutants. Experiment performed with three independent bacterial cultures with means and SEs plotted. (**C**) Relative expression of *K. pneumoniae bamA* (black bars) and *bamK* (gray bars) in PTB1-1-resistant *baeS* mutants measured by qRT-PCR (normalized to *rpoD* and parental wild-type *K. pneumoniae* expression levels). Experiment performed with three independent bacterial cultures with means and SEs plotted. (**D**) Expression of P*_bamK_*-GFP transcriptional reporter in wild-type (solid black line) and *baeS*^V295G^ mutant (solid blue line) strains measured by flow cytometry. A vector control strain lacking a reporter is shown as a dashed gray line. Two technical replicates of biological duplicates were run for each strain, and representative traces are shown.

Because BaeS, RamR, and OqxR are all predicted to regulate gene expression (31–34), RNA-seq analysis was performed on representative resistant strains with single mutations in each gene: *baeS*^V295G^, *ramR*^A72fs^ (*ramA* with a frameshift at position 72), and *oqxR*^A19V^. Of note, no growth defects were observed for the PTB1-1-resistant *K. pneumoniae* mutant strains (Fig. S2A). In the *K. pneumoniae baeS*^V295G^ mutant, 41 genes were upregulated and 88 genes were downregulated compared to the wild-type *K. pneumoniae* parent strain (|log_2_FC| ≥ 1 and FDR ≤ 0.05), including known members of the BaeSR regulon, for example, *spy* (log_2_FC = 6.1, FDR = 1.8e−16) and *mdtA* (log_2_FC = 3.7, FDR = 1.3e−13) (Fig. 2A). There were 123 upregulated and 58 downregulated genes in the *K. pneumoniae ramR*^A72fs^ mutant and 61 upregulated and 23 downregulated genes in the *K. pneumoniae oqxR*^A19V^ mutant compared to the wild-type *K. pneumonia*e parent strain (|log_2_FC| ≥ 1 and corrected FDR ≤ 0.05) (Fig. 2A). Principal component and pairwise correlation analyses of the expression profiles of genes differentially expressed in at least one mutant compared to the wild-type parent strain show distinct expression among mutant strains (Fig. S2B and C). None of the resistant mutants exhibited altered expression of genes encoding the BAM complex: *bamA*, *bamB*, *bamC*, *bamD*, or *bamE* (Fig. S2D). Notably, each mutant exhibited increased expression of a different known efflux pump system (Fig. S2E); however, the deletion of these efflux pumps in the PTB1-1-resistant mutant background did not restore sensitivity to the antibacterial macrocycle (Table S4).

### The BaeSR two-component signal transduction system regulates *bamK* expression

To identify alternative mechanisms of resistance, we focused on the expression of the PTB1-1 target, BamA, and its recently described paralog, BamK (28). BamK is found among *Klebsiella* species, possesses OMP folding activity, and can functionally replace BamA to support the growth of a Δ*bamA* mutant strain (28). Moreover, sequence analysis reveals that the structurally determined PTB1-1-binding site on BamA is divergent from the corresponding site on BamK based on homology (Fig. 1C and Fig. S3A). Although none of the PTB1-1 resistant mutants exhibited altered expression of any component of the BAM complex (*bamA*, *bamB*, *bamC*, *bamD*, and *bamE*), *bamK* was upregulated in the *K. pneumoniae baeS*^V295G^ mutant compared to the wild-type *K. pneumoniae* parent (log_2_FC = 4.0, FDR = 2.1e−12) (Fig. 2A and B, and Fig. S2D). We focused on this mutant to determine if *bamK* expression led to PTB1-1 resistance.

BaeS^V295G^ constitutively activates BaeR, resulting in derepression of genes important for the membrane-stress response (35). Thus, the *baeS*^V295G^ mutant can provide insight into the role and regulation of *bamK* in *K. pneumoniae*. Six distinct *baeS* mutations were identified in different PTB1-1 resistant isolates (Table S3). Quantitative, real-time PCR analysis (qRT-PCR) of four of the *baeS* mutants revealed that *bamK* expression increased 30- to 850-fold, depending on the mutant, compared to the wild-type parent strain while *bamA* levels remained unchanged (Fig. 2C). To validate the role of BaeS in *bamK* expression, we constructed a *bamK* promoter fusion to green fluorescent protein (GFP) (P*_bamK_*-GFP). Similar to the RNA-seq and qRT-PCR observations, GFP signal was only observed in the *baeS*^V295G^ mutant and not in a wild-type *K. pneumoniae* background (Fig. 2D and Fig. S4). Examination of the DNA sequence upstream of the *bamK* coding region (28) revealed a sequence that is nearly identical to the consensus BaeR binding motif in *E. coli* (36) (Fig. S3B). Although these observations are consistent with a role for BaeSR in the regulation of *bamK*, additional investigations will be required to understand the molecular mechanism. Thus, PTB1-1-resistant mutations in *baeS* increased *bamK*, but not *bamA*, expression.

### BamK production provides resistance to BamA-targeting macrocycles

To determine if *bamK* expression was sufficient to impart PTB1-1 resistance in the *baeS* mutants, we constructed *E. coli bamA* conditional mutants complemented with plasmids constitutively expressing either *E. coli bamA* (*bamA-Ec*), *K. pneumoniae bamA* (*bamA-Kp*), or *K. pneumoniae bamK* (*bamK-Kp*). In these strains, chromosomal expression of *bamA* is controlled by arabinose induction such that in the absence of arabinose, only *bamA* encoded on the complementing plasmid is expressed. As expected, the *E. coli bamA* conditional mutant with an empty vector did not grow in the absence of arabinose (see Fig. 4A). However, the *E. coli bamA* conditional strain was able to grow in the absence of arabinose when a plasmid constitutively expressing *bamA-Ec, bamA-Kp*, or *bamK-Kp* was present (Fig. 4A), confirming that *K. pneumoniae* BamA and *K. pneumoniae* BamK can functionally substitute for endogenous BamA in *E. coli* (28). Importantly, the *E. coli bamA* conditional strains expressing *bamA-*Ec or *bamA-*Kp remained sensitive to PTB1-1; however, the expression of *bamK-*Kp resulted in PTB1-1 resistance with an MIC >100 µM (Table 3). Thus, BamK is insensitive to inhibition by PTB1-1, consistent with the lack of homology in the PTB1-1 binding site (Fig. 1C and Fig. S3A), and the expression of *bamK* alone in *E. coli* was sufficient to support growth and provide resistance to this BamA-targeting antibacterial macrocyclic peptide.

**TABLE 3:** Minimal inhibitory concentrations (MICs) for the BamA-targeting antibacterial macrocycle PTB1-1 against *E. coli* MG1655 and *K. pneumoniae* ATCC 43816 strains expressing *E. coli bamA*, *K. pneumoniae bamA*, or *K. pneumoniae bamK*^*a*^

Bacterial strain	Plasmid	PTB1-1 MIC (µM)
*E. coli* MG1655 conditional *bamA*	VC	No growth
*E. coli* MG1655 conditional *bamA*	p-*bamA*-Ec	0.39, 0.78
*E. coli* MG1655 conditional *bamA*	p-*bamA*-Kp	0.78, 1.56
*E. coli* MG1655 conditional *bamA*	p-*bamK*-Kp	>100
*K. pneumoniae* ATCC 43816	None	6.25
*K. pneumoniae* ATCC 43816 Δ*bamK*	None	6.25
*K. pneumoniae* ATCC 43816 BaeS^V295G^	None	>100
*K. pneumoniae* ATCC 43816 BaeS^V295G^ Δ*bamA*	None	>100
*K. pneumoniae* ATCC 43816 BaeS^V295G^ Δ*bamK*	None	6.25

^*a*^ Strains were grown in LB media.

**Fig 3:**
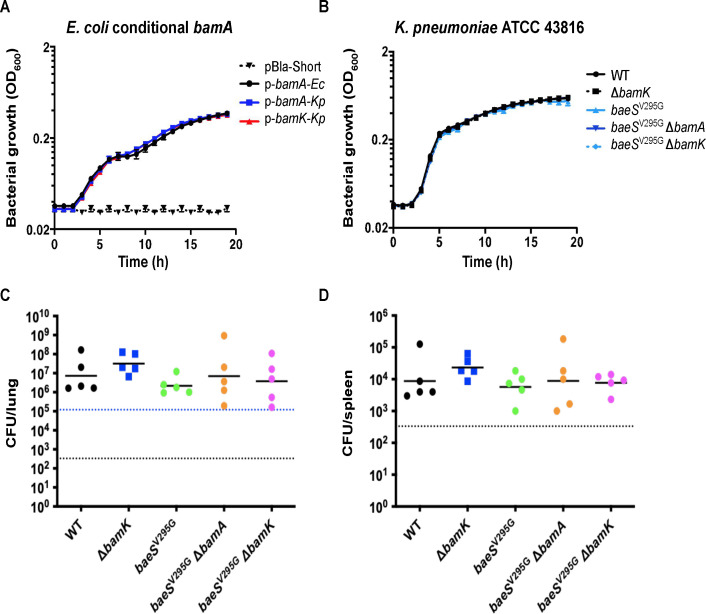
Growth of *bamA* and *bamK* deleted and complemented strains of *E. coli* MG1655 and *K. pneumoniae* ATCC 43816. (**A**) Bacterial growth (measured by OD_600_) over time was assessed from bacterial cells initially grown overnight in the presence of arabinose. This was followed by subculturing for 2 h without arabinose and diluting *E. coli* conditional *bamA* strains without the arabinose inducer. The strains were complemented with plasmids that constitutively express either *E. coli bamA* (*bamA-Ec*, solid black line), *K. pneumoniae bamA* (*bamA-Kp*, solid blue line), *K. pneumoniae bamK* (*bamK-Kp*, solid red line), or an empty vector (VC, dashed line). The means and standard errors (SEs) are plotted for triplicate experiments. (**B**) Bacterial growth (measured by OD_600_) over time of *K. pneumoniae* Δ*bamA* and Δ*bamK* deletion strains in wild-type or *baeS*^V295G^ mutant backgrounds. Means and SEs are plotted for triplicate experiments. Bacteria (CFUs) recovered from the (**C**) lungs and (**D**) spleen of Balb/c mice 24 h after intranasal infection with 2 × 10^5^ CFU of indicated *K. pneumoniae* mutant strains. Inocula (dashed blue line in panel **C**) and the limit of detection (dashed black lines in panels **C** and **D**) are indicated. Five mice were infected in each test group.

We next sought to determine if *bamK* expression was necessary for PTB1-1 resistance in *K. pneumoniae*. Consistent with the lack of *bamK* expression in the wild-type strain, the deletion of *bamK* from wild-type *K. pneumoniae* did not affect sensitivity to PTB1-1 (Table 3). In contrast, the removal of *bamK* in the *K. pneumoniae baeS*^V295G^ strain, which exhibited >250-fold increased *bamK* expression (Fig. 2C), reduced the PTB1-1 MIC to that observed for the wild-type *K. pneumoniae* strain (6.25 µM) (Table 3), demonstrating *bamK* was necessary for PTB1-1 resistance in the *baeS*^V295G^ mutant background. Remarkably, we were able to delete *bamA*, an essential gene, in the *baeS*^V295G^ mutant background, and this *K. pneumoniae baeS*^V295G^ Δ*bamA* mutant grew at the same rate as the parent strain (Fig. 3B) and remained resistant to PTB1-1 (Table 3). Importantly, while it was possible to generate both the *K. pneumoniae baeS*^V295G^ Δ*bamA* and the *K. pneumoniae baeS*^V295G^ Δ*bamK* double mutants at high frequencies, our attempts to delete *bamA* in a *baeS*^V295G^ Δ*bamK* mutant to generate the *K. pneumoniae baeS*^V295G^ Δ*bamK* Δ*bamA* triple mutant failed to result in colonies after three independent attempts. In total, our results suggest that BamA and BamK are functionally redundant OMP foldases, and the presence of at least one is essential for cell viability.

To determine whether the activation of *bamK* by *baeS*^V295G^ was sufficient to support growth *in vivo*, we infected mice with these *K. pneumoniae* mutant strains. Twenty-four hours after intranasal infection of a Balb/c pneumonia model, there was no significant difference in the colony forming units (CFUs) recovered among *K. pneumoniae* Δ*bamK*, *K. pneumoniae baeS*^V295G^, *K. pneumoniae baeS*^V295G^ Δ*bamK, K. pneumoniae baeS*^V295G^ Δ*bamA*, and wild-type *K. pneumoniae* infected lungs or spleens (Fig. 3C and D). Importantly, the *in vivo* essentiality of BamA is supported by the finding that a BamA-selective natural product inhibitor, darobactin, is efficacious in a mouse infection model (6). Our findings indicate that while OMP foldase activity is necessary for *K. pneumoniae* viability, *bamA* is not essential when *bamK* expression is induced by the BaeSR system *in vitro* or *in vivo*. Therefore, *K. pneumoniae* possesses a natural strategy to entirely circumvent the BamA-targeting antibacterial macrocyclic peptide PTB1-1 through BaeSR-mediated upregulation of *bamK*.

### Divergence of the extracellular surface of BamA and BamK influences antibody and bacteriophage binding

**Fig 4:**
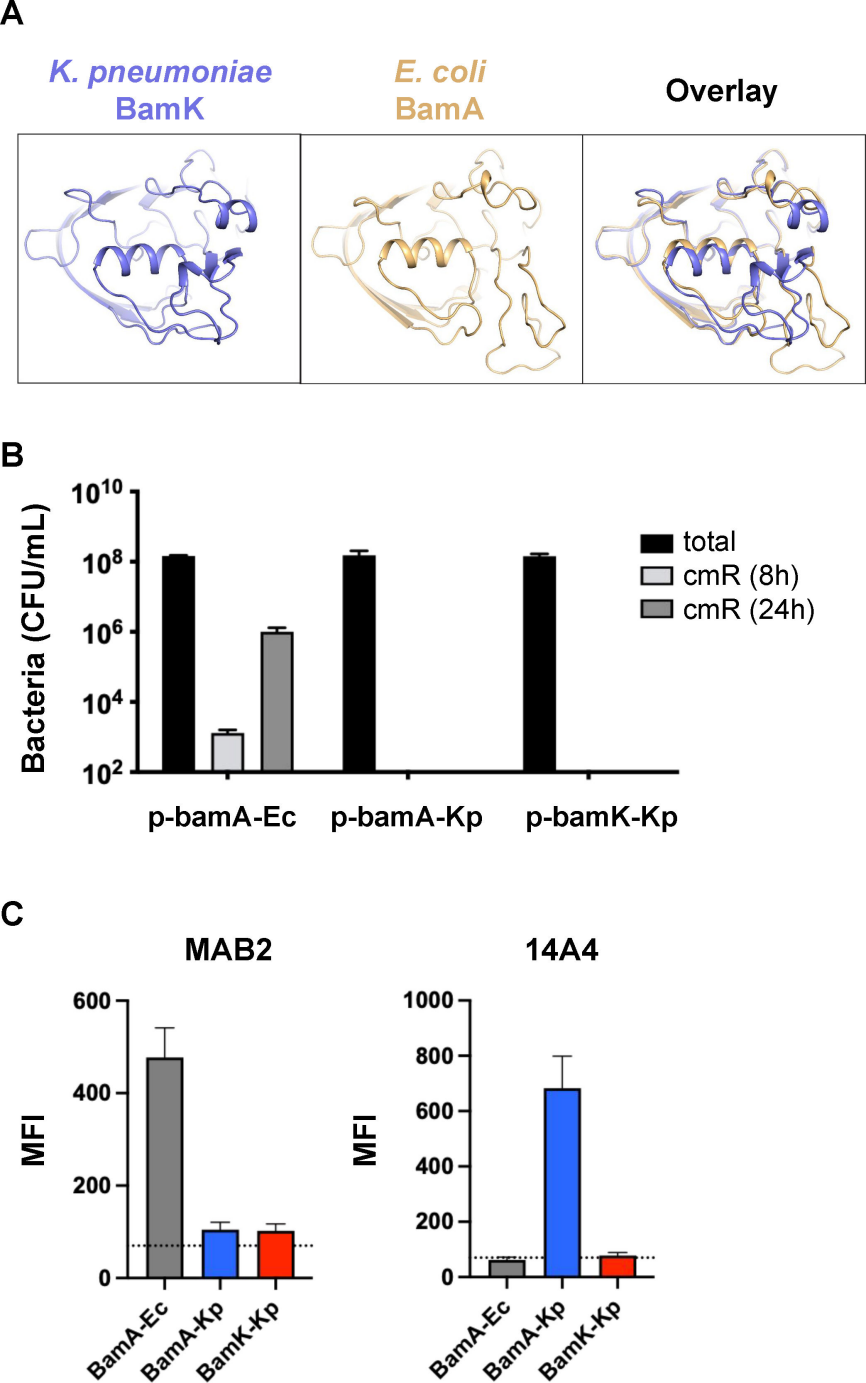
Bacteriophage and antibody interactions with BamA and BamK on cells. (**A**) Structural comparison of the predicted structures of the extracellular loops of *K. pneumoniae* BamK (left, blue), *E. coli* BamA (middle, gold), and an overlay of the two structures (right). (**B**) Bacterial growth (CFU/mL) of *E. coli* MG1655 conditional *bamA* strain in the absence of arabinose inducer constitutively expressing *E. coli bamA* (BamA-Ec), *K. pneumoniae bamA* (BamA-Kp), or *K. pneumoniae bamK* (BamK-Kp) 8 h (light gray bars) or 24 h (dark gray bars) after infection with 933Δ*stx2::cm* bacteriophage on nonselective LB (total) (black bars) or LB plus chloramphenicol (cm) (gray bars). Means and SEs for triplicate experiments show the total number of bacteria (growth on LB) and bacteria infected with the 933Δ*stx2::cm* (growth on LB plus cm). (**C**) Binding of monoclonal anti-BamA antibodies MAB2 (9) (left) and 14A4 (38) (right) to an *E. coli* MG1655 conditional *bamA* strain in the absence of arabinose inducer constitutively expressing *E. coli bamA* (BamA-Ec, gray bars), *K. pneumoniae bamA* (BamA-Kp, blue bars), or *K. pneumoniae bamK* (BamK-Kp, red bars) measured by flow cytometry (mean fluorescence intensity, MFI). Means and SEs for triplicate experiments are plotted.

BamA possesses long extracellular loops that are divergent in sequence even between closely related gram-negative bacteria (37), suggesting the possibility that there is evolutionary pressure to diversify the exposed regions of this essential protein. Indeed, PTB1-1 exhibits differential activity across even closely related species, likely due to divergence of its extracellular binding site on BamA (Table 1). For BamK, the extracellular loops are divergent in sequence and structure when compared to BamA (Fig. 4A and Fig. S3A), and moreover, the PTB1-1 binding site residues on BamA lack homology compared to the corresponding positions on BamK, providing a rationale for PTB1-1 resistance (Fig. 1C). Thus, any molecule, synthetic or natural, that targets accessible structural features of BamA could encounter a distinct site on BamK, and this could prevent inhibition of an essential process in *K. pneumoniae*.

In addition to being a target for small molecule inhibitors, BamA is a receptor for bacteriophages, including the Stx2 short-tailed bacteriophage (39). We infected *E. coli* conditional *bamA* strains complemented with *bamA*-Ec, *bamA*-Kp, or *bamK*-Kp with Stx2 933W bacteriophage encoding an antibiotic resistance marker to evaluate bacteriophage infection. The Stx2 933W bacteriophage only infected bacterial cells expressing *bamA*-Ec but not *E. coli* expressing *bamA*-Kp or *bamK*-Kp (Fig. 4) suggesting differences in the BamA extracellular loops between these species were sufficient to prevent infection by Stx2 933W.

Due to their localization, the extracellular loops of BamA can also be targeted by antibodies. Discovery campaigns have generated antibodies that distinguish BamA proteins from closely related species, such as *E. coli* vs *K. pneumoniae* (9, 38). The monoclonal antibody MAB2 (9) only bound to *E. coli* BamA and was unable to bind BamA-Kp or BamK-Kp when either was produced in an LPS-truncated *E. coli* Δ*waaD* mutant strain (Fig. 4). This result also confirms the absence of native BamA in the Δ*bamA* mutant. While antibodies that bound BamA-Kp were rare (9, 38), 14A4 was able to distinguish BamA-Kp from both BamA-Ec and BamK-Kp. This antibody specifically recognized *E. coli* Δ*waaD* expressing *bamA*-Kp, but not *bamA*-Ec or *bamK*-Kp, highlighting the divergent extracellular sequences of these BamA paralogs. These results suggest a potential role for BamK in avoiding detection by host immune response and bacteriophages that target BamA.

## DISCUSSION

Two-component systems enable gram-negative bacteria to sense stress and regulate gene expression accordingly. In the canonical BaeSR two-component system, BaeS auto-phosphorylates in response to an environmental signal and subsequently phosphorylates the response regulator BaeR, a transcription factor. Here, we report that BaeSR controls the expression of *bamK*, a *bamA* paralog, in *K. pneumoniae*. Specifically, *baeS* mutants enable cells to survive in the presence of a potent and selective antibacterial BamA inhibitor PTB1-1.

Signals that activate BaeS are known to cause stress in the envelope of gram-negative bacteria, such as misfolded pilus subunits and metal toxicity (31, 40). In response to these stressors, BaeR upregulates genes involved in mitigating stress, including the periplasmic chaperone Spy, and the multidrug efflux pump Mdt. The inclusion of *bamK* in the BaeSR regulon suggests that this *bamA* paralog is induced under envelope-stress conditions, which can be rationalized in a number of ways. It is possible that increasing the folding and insertion of OMPs in *K. pneumoniae* by increasing the total number of BAM complexes, with BamA or BamK, could alleviate envelope stresses. Alternatively, envelope stresses could compromise the activity of BamA, and BamK might function better under some yet-to-be determined environmental conditions. In any case, the ability to induce expression of *bamK* under these conditions could allow *K. pneumoniae* to overcome otherwise detrimental stress conditions. Future efforts aimed at identifying conditions that lead to induction of *bamK* will be important for understanding the physiological role of BamK.

In addition to a potential role in mitigating envelope stress, BamK could also provide a survival strategy for *K. pneumoniae* if BamA activity is compromised. Our selection used a synthetic macrocyclic peptide, PTB1-1, that inhibits BamA but is unable to interfere with BamK, thus rationalizing resistance through *bamK* upregulation. In addition to PTB1-1 and other synthetic inhibitors of BamA, antibacterial natural products, specifically darobactin and dynobactin, target BamA, suggesting *bamK* induction could be a relevant strategy in nature (6, 8, 25, 26, 41). BamA is also a target for antibodies and bacteriophage. Though a direct antibacterial role for antibodies against BamA in the host response has not been described, BamA monoclonal antibodies can differentiate BamA at the species level, revealing extracellular epitopes that are unique to *E. coli*, *K. pneumoniae*, or *Enterobacter cloacae* (38). BamA-targeting antibodies produced by the immune system against *K. pneumoniae* would be unlikely to also interact with BamK given the low homology (62.7% identity), especially in the extracellular loop regions accessible to antibodies (Fig. S3A). Similarly, bacteriophage can use BamA as a receptor and the distinct sequence of BamK provides *K. pneumoniae* with a potential strategy to avoid infection. Finally, BamA is the target for contact-dependent inhibition, a toxin delivery mechanism used by bacteria that requires direct cell-to-cell contact (42). It remains to be seen whether the expression of *bamK* can provide resistance to contact-dependent inhibition and other natural inhibitory mechanisms targeting BamA and why *Klebsiella* species, but not all gram-negative bacteria including *E. coli*, encode BamK, but future investigation into natural environments that induce *bamK* should provide important insight into these questions.

BamA has received recent attention as a potential novel antibiotic target. Small molecules such as MRL-494, natural products such as darobactin and dynobactin, chimeric peptidomimetics of murepavadin and polymyxin variants, macrocyclic peptides like PTB1-1, and monoclonal antibodies have all been demonstrated to interfere with the essential process of OMP folding (6–9, 11, 25–27, 41). As these and other discovery efforts continue, the impact of BamK will need to be considered in order to ensure activity against *K. pneumoniae* and other *Klebsiella* species where this paralog could provide resistance. Additionally, the mechanism of resistance imparted by mutations in *ramR* and *oqxR* that were described here necessitate further exploration. Continued investigations into BamA modulators as well as exploring OMP folding in diverse gram-negative species promises to provide important insight into these fundamental biological questions.

## MATERIALS AND METHODS

### Strain construction, growth conditions, and growth curves

Bacterial strains and relevant primers are listed in Table S5. Mutant strains were created using λ Red recombination (43, 44). Briefly, pKD4 or pKD3 was amplified with primers containing 50 bp nucleotide homology extensions to the gene of interest. *Klebsiella* transformation efficiency was improved by protecting linear pieces of DNA from nuclease degradation by adding phosphorothioate bonds (Integrated DNA Technologies) to the primers. The linear product was transformed into the appropriate background strain containing pSIM18 (45), recovered for 4 h, and selected on media containing 50 μg/mL kanamycin or 12.5 μg/mL chloramphenicol, as appropriate. Mutants were confirmed by PCR amplification of the locus with primers annealing upstream and downstream of the target gene (Table S5) and visualized by on agarose gels to confirm size shifts. Additionally, PCR products were analyzed by Sanger sequencing to confirm expected deletion of the target gene (primers listed in Table S5).

Bacterial cultures were grown at 37°C in Luria-Bertani (LB [Miller], Sigma-Aldrich L3522) or Mueller Hinton II cation-adjusted broth (MHB II, BBL 212322) supplemented with 0.002% Tween-80 prepared according to manufacturer’s instructions. When appropriate, media was supplemented with kanamycin (50 μg/mL), carbenicillin (50 μg/mL), chloramphenicol (12.5 μg/mL), hygromycin (200 μg/mL), gentamicin (10 μg/mL), or arabinose (0.2% wt/vol) as indicated.

Bacterial growth curves were performed in LB media at 37°C without agitation. Bacterial cells pre-grown to log phase (OD_600_ 0.4–0.6) were inoculated to a starting OD_600_ 0.01. At each time point, bacterial growth was measured by optical density at OD_600_. Quadruplicate cultures were run. For strains conditionally expressing *bamA*, cells were grown overnight in the presence of arabinose. The spent media was then removed, and the cells were diluted to an OD_600_ of 0.01 in LB medium without arabinose, followed by subculturing for 2 h. The cells were then inoculated and monitored as previously described. Biological replicates refer to individual colonies inoculated into separate tubes for overnight growth, followed by back-dilution to grow to mid-log, prior to inoculating the individual well for the growth curve.

### Minimal inhibitory concentration assays

MICs were determined by performing twofold serial dilutions of peptides in LB or MHB II broth supplemented with 0.002% Tween-80 as indicated in the figure legends to a final volume of 0.1 mL in round-bottom 96-well assay plates (Corning Life Sciences No. 3788). Peptides were initially resuspended in 100% DMSO to 10 mM and subsequently diluted in the relevant growth medium to the appropriate concentration. Each well was inoculated with 5 × 10^5^ CFU/mL of the screening stain and incubated at 37°C without agitation for 18 h. Plates were scored by eye, and the lowest compound concentration preventing visible growth defined the MIC. A minimum of two biological replicates were performed.

Bacterial strains were prepared from log-phase cells cultured in the specified growth media. For strains conditionally expressing *bamA*, cells were grown overnight with arabinose. After removing the spent media, the cells were diluted to an OD_600_ of 0.01 in LB medium without arabinose and then subcultured for 2 h. For strains carrying a plasmid, cells were grown overnight under antibiotic selection. The spent media was removed, and the cells were diluted to an OD_600_ of 0.01 in LB medium without antibiotics, followed by 2 h of subculturing. MICs were tested without additional antibiotics.

### Frequency of resistance and on-target *bamA* sequencing

Log-phase bacterial cells were diluted to 1–5 × 10^7^ CFU/mL in the indicated liquid medium to a final volume of 0.2 mL containing 2×–4× MIC of each peptide in round-bottom 96-well assay plates (Corning Life Sciences No. 3788). A minimum of 300 wells were monitored for bacterial growth after incubation at 37°C without agitation for 2 days. Selections were performed using ratios of bacterial cells to peptide concentration such that <5% of the wells exhibited growth, minimizing the chance that wells exhibiting growth contained more than one resistant isolate. Isolated spontaneous resistant colonies were obtained from wells with bacterial growth and streak purified once for additional colony isolation. A single colony was selected, and resistance was confirmed. Freqency of resistance (FOR) for each strain and peptide was determined by dividing the number of wells that regrew after 2 days at 37°C by the initial viable cell count.

For mutants with an MIC >2-fold above the parent strain, the *bamA* gene was analyzed by Sanger sequencing. Briefly, colony PCR was performed (46) and *bamA* was amplified with primers “*bamA* Kp outside F” and “*bamA* Kp outside R” using GoTaq DNA polymerase (Promega). Samples were Sanger sequenced by Elim Biopharm with primers spanning the entire *bamA* sequence (Table S5).

### Resistant mutant whole genome sequencing and variant analysis

Genomic DNA was isolated from PTB1-1-resistant bacterial cultures as well as the parent strains (*E. coli* ATCC 25922, *K. pneumoniae* ATCC 43816, and *K. pneumoniae* ATCC 700721) as specified for gram-negative bacteria using the DNeasy Blood and Tissue kit (Qiagen) and sequenced by HiSeq 2000 (Illumina) to generate 75-bp paired-end data. Reads from each isolate were mapped onto the reference genome (*E. coli* ATCC 25922, accession number NZ_CP009072 and *K. pneumoniae* ATCC 43816, accession number NZ_CP064352) using GSNAP version 2013-10-10 (47) with default parameter settings. Candidate variants were identified using the Genentech in-house bioinformatics pipeline, which utilizes the following R and Bioconductor packages: GenomicRanges (48), GenomicAlignments (48), VariantTools (49), and gmapR (43). Only base-calls with a Q-score ≥ 30 were tallied for variant calling.

### RNAseq analysis and cell preparation

Bacterial cells were grown in LB media with agitation at 37°C. Overnight cultures were inoculated to a starting OD_600_ 0.01 and grown to log phase (OD_600_ 0.4–0.6). Cells were back-diluted a second time to a starting OD_600_ 0.01 and grown to log phase (OD_600_ 0.4). Bacterial cultures were treated with RNAprotect (Qiagen). RNA was purified with the RNeasy kit (Qiagen), including the on-column DNase I treatment.

Total RNA was quantified with Qubit RNA HS Assay Kit (Thermo Fisher Scientific), and quality was assessed using RNA ScreenTape on TapeStation 4200 (Agilent Technologies). Sequencing libraries were generated from 400 ng of total RNA using the TruSeq Stranded Total RNA Kit (Illumina catalog# 20020596) in combination with the Illumina Ribo-Zero Plus rRNA Depletion Kit (Illumina catalog# 20037135). Libraries were quantified with Qubit dsDNA HS Assay Kit (Thermo Fisher Scientific), and the average library size was determined using D1000 ScreenTape on TapeStation 4200 (Agilent Technologies). Libraries were pooled and sequenced on NovaSeq 6000 (Illumina) to generate 30 million single-end 50-base-pair reads for each sample.

The fastq sequence files for all RNA-seq samples were aligned to the *K. pneumoniae* ATCC 43816 reference genome using the GSNAP (47). Only uniquely mapped reads were used for further analysis. The Bioconductor package, HTSeqGenie, was used to obtain read counts and RPKMs per gene. Differential expression analysis was performed with voom+limma (50).

### Quantitative real-time PCR

The manufacturers’ instructions were followed for each step unless otherwise noted. Mid-log (OD_600_ 0.4) bacterial cultures were treated with RNAprotect (Qiagen). RNA was purified with the RNeasy kit (Qiagen) including the on-column DNAse I treatment. cDNA was created using the High-Capacity cDNA Reverse Transcription Kit (Applied Biosystems). qRT-PCR was performed on 7500 Real-Time PCR System (Applied Biosystems) using Power SYBR Green PCR Master Mix (Applied Biosystems) according to manufacturer’s instructions. Relative gene expression was calculated using a 2^−ΔΔCT^ method (51) with *rpoD* serving as the reference gene.

### Bacteriophage transductions

Preparation of 933W recombinant chloramphenicol-resistant phage (*E. coli* O157:H7 ATCC 43895 (933WΔ*stx2::cat*)) was performed similar to previously described methods (52). Briefly, bacterial cultures were grown in tryptic soy broth (TSB) supplemented with 5 mM CaCl_2_ to mid-log (OD_600_ 0.5). Mitomycin C was added to a final concentration of 0.5 μg/mL, and the cultures were incubated overnight at 37°C. After incubation, the cultures were centrifuged for 1 min at 14,500 × *g* and supernatants filtered through low-protein-binding 0.22-μm-pore-size membrane filters (Millipore). The phage suspensions were added at a 1:4 dilution to mid-log (OD_600_ 0.3) bacterial cultures of the target strain and incubated for 8 h or 24 h. CFUs were enumerated on LB agar plates with and without chloramphenicol (12.5 µg/mL).

### Reporter assay by flow cytometry

The *bamK* reporter construct was made in pET-28a (Addgene). The predicted intergenic region upstream of *bamK* was cloned upstream of the gene encoding Dasher GFP. Bacterial strains were grown to log phase in LB. Cells were harvested and re-suspended to an OD_600_ 0.5 in wash buffer (PBS supplemented with 1% BSA) and fixed in 2% paraformaldehyde (PFA) in PBS for 10 min prior to analysis on a FACSAria (BD) using FACSDiva software (BD). Ten thousand events were captured for analysis and quantified for mean fluorescence intensity (MFI). Technical duplicates of two biological replicates were run for each strain and representative traces are shown.

### *In vivo* experiments

Bacterial strains were prepared fresh from mid-log (OD_600_ 0.5) cultures grown in LB, washed 1× in PBS, and resuspended to ~4 × 10^6^ CFU/mL. Seven- to eight-week-old Balb/c mice (Charles River Laboratory, Hollister, CA) were infected intranasally with 50 µL of each indicated *K. pneumoniae* strain at 2 × 10^5^ CFUs per mouse. *K. pneumoniae* CFUs in the lungs and spleens were determined 24 h post-infection by serial dilutions on LB agar plates.

### Structural modeling of *K. pneumoniae* BamA and BamK

The model of *K. pneumoniae* BamA (BamA-Kp, AF-A0A378C0J7-F1) was downloaded from the AlphaFold Protein Structure Database (https://alphafold.ebi.ac.uk/) (53, 54). The model of *K. pneumoniae* BamK was generated using an in-house installation of DeepMind AlaphaFold2 v2.3.2 available on GitHub (https://github.com/google-deepmind/alphafold/releases/tag/v2.3.2) (53).

## Data Availability

All data supporting the findings of this study are available. DNA and RNA sequencing data have been deposited in the Sequence Read Archive of the National Center for Biotechnology Information under BioProject ID PRJNA1425164.

## SUPPLEMENTAL MATERIAL

Supplemental Information mbio.03497-24-s0001.pdf Supplemental figures and tables

Source Data mbio.03497-24-s0002.xlsx Source data for all figures.

ASM does not own the copyrights to Supplemental Material that may be linked to, or accessed through, an article. The authors have granted ASM a non-exclusive, world-wide license to publish the Supplemental Material files. Please contact the corresponding author directly for reuse.
