# Supplementary material for: The BaeSR two-component system activates bamK, a paralog of the essential β-barrel foldase bamA, in Klebsiella pneumoniae
Source: mBio. 2026 Mar 23;17(4):e03497-24. doi: 10.1128/mbio.03497-24 (PMC13059784; doi:10.1128/mbio.03497-24)
Supplement: Supplemental Information — Supplemental figures and tables [file mbio.03497-24-s0001.pdf]

Supplemental Information for:

A two-component system activates *bamK*, a homolog of the essential β-barrel foldase *bamA*, in *Klebsiella pneumoniae*

Kelly M. Storek[1*], Janina Reeder[2+], Dawei Sun[3+], Donghong Yan[4], Austin K. Murchison[1,5], Min Xu[4], Elizabeth Skippington[2], Steven T. Rutherford[1*]

[1]Department of Infectious Diseases, Genentech, Inc., CA, USA
[2]Department of Bioinformatics, Genentech, Inc., CA, USA
[3]Department of Structural Biology, Genentech, Inc., CA, USA
[4]Department of Translational Immunology, Genentech, Inc., CA, USA
[5]Current: Department of Chemical Engineering, Stanford University, CA, USA

[+]Authors contributed equally

*Address correspondence to Kelly M. Storek (storek.kelly@gene.com) and Steven T. Rutherford (rutherford.steven@gene.com)

CONTENTS **PAGES**

SI Fig. S1-S3………………………………………………………… 2-7
SI Tables S1-S5……………………………………………………… 8-17
SI Reference………………………………………………………… 18

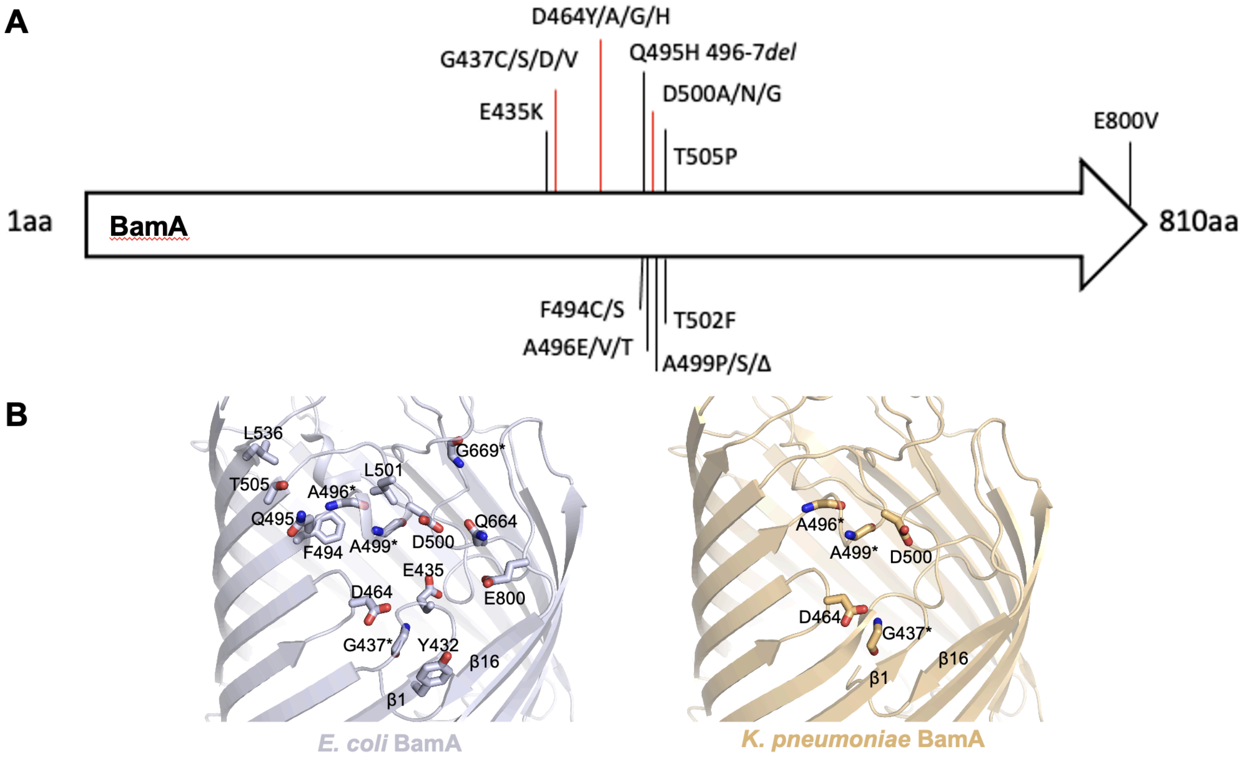

SI Fig. S1. Amino acid substitutions and deletions in BamA identified by selections for PTB1-1 resistance. (**A**) Approximate positions of substitutions in BamA that exhibited >4-fold increased PTB1-1 MICs identified in *E. coli* and *K. pneumoniae* are shown. Black lines are positions identified only in *E. coli* and red lines are substitutions selected in both *E. coli* and *K. pneumoniae*. (**B**) Structural models of *E. coli* BamA (left, light blue, PDB: 9CNX) and *K. pneumoniae* BamA (right, tan, AF-A0A378C0J7-F1) with amino acid substitutions leading to PTB1-1 resistance shown as sticks and labeled.

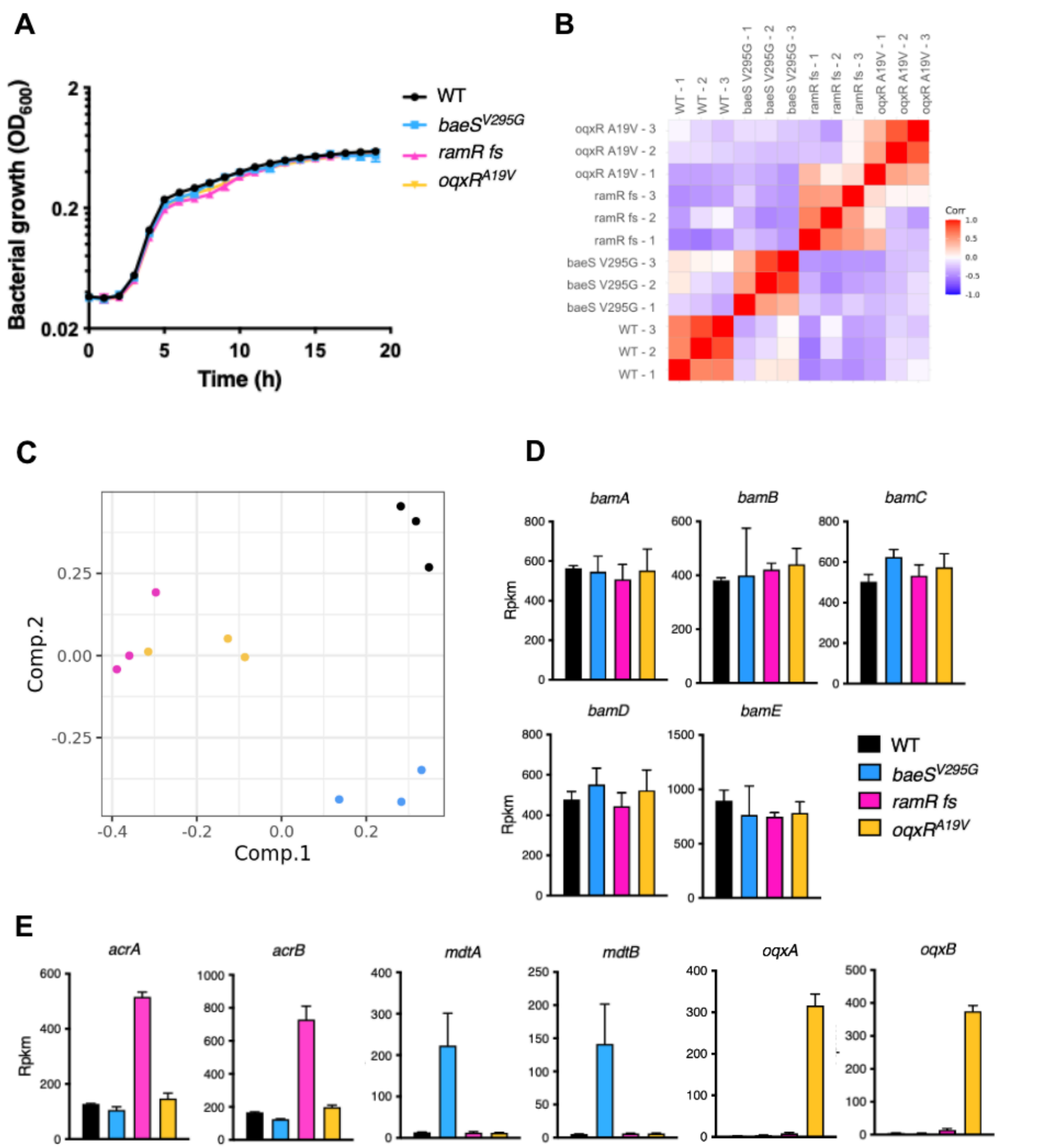

SI Fig. S2. Analysis of *K. pneumoniae* PTB1-1 off-target resistance mutants. (**A**) Growth curves of PTB1-1-resistant *K. pneumoniae* mutants in LB media measured by OD_{600} over time. The parent wild-type (WT) is *K. pneumoniae* ATCC 43816. Experiments were performed in quadruplicate and the means and standard errors (SEs) are plotted. (**B**) Heat map showing pairwise correlations of gene expression profiles for genes satisfying |Log2FC| ≥ 1 and p values ≤ 0.05 in at least one of the three contrasts shown in **Fig. 2A**. (**C**) Principal component analysis of RNA-seq data for differentially expressed

genes identified in **Fig. 2A**. Expression (Rpkm, reads per kilobase per million mapped reads) of genes encoding (**D**) the BAM machinery (*bamA*, *bamB*, *bamC*, *bamD*, and *bamE*) and (**E**) individual efflux pump components (*acrAB*, *mdtAB*, and *oqxAB*) differentially expressed in each *K. pneumoniae* PTB1-1-resistant mutant. Means and standard deviations for triplicate RNA-seq experiments are shown.

A

```
                   L1                                   L2
BamA-Ec   RNTGSFNFGIGYGTESGVSFQAGVQQDNWLGTGYAVGINGTKNDYQTYAELSVTNPYFTV480
BamA-Kp   RNTGSFNFGIGYGTESGVSFQAGVQQDNWLGTGYAVGINGTKNDYQTYTELSVTNPYFTV480
BamK-Kp   RNTGSFNVGLGFGTDSGVSYQLGVTQDNWLGTGNSVSFNGTRNSYQSYLELGATNPWFTV480
          *******.*:*:**:****:* ** ******** :*.:***:*.**:* **..***:***

                     L3                                        L3
BamA-Ec   DGVSLGGRLFYNDFQADDADLSDYTNKSYGTDVTLGFPINEYNSLRAGLGYVHNSLSNMQ540
BamA-Kp   DGVSLGGRVFYNDFDANDADLSDYTNKSYGTDITLGFPVNEYNTLRAGVGYVHNSLSNMQ540
BamK-Kp   DGISLGGKIFYNSYDASDADAGSYNQQSYGLGSTLGFPISENNSLNLGLDYVHNRLTNMD540
          **:****::***.::*.*** ..*.::*** . *****:.* *:*. *:.**** *:**:

BamA-Ec   PQVAMWRYLYSMGEHPSTSD----QDNSFKTDDFTFNYGWTYNKLDRGYFPTDGSRVNLT596
BamA-Kp   PQVAMWRYLNSMGQYPDNTN----DRNSFSANDFTFNYGWTYNKLDRGFFPTEGSRVNLN596
BamK-Kp   PELTTWRYLSSRGIEPSVVTKDGDSGAKYSANDYFVSLGWGYNDLDRGFFPRAGNKSSLS600
          *::: **** * *  *.        .  .:.::*: .. ** **.****:**  *.: .*.

          L5                                            L6
BamA-Ec   GKVTIPGSDNEYYKVTLDTATYVPIDDDHKWVVLGRTRWGYGDGLGGKEMPFYENFYAGG656
BamA-Kp   GKVTIPGSDNEYYKATLDTATYVPIDNDHQWVVLGRTRFGYGDGIGGKEMPFYENFYAGG656
BamK-Kp   GKVTLPGSDNSYYKLSFDTAQYLPLSENKRWVWMERLRAGYAGGLDGKSVPFYDNFYAGG660
          ****:*****.*** ::*** *:*:.::::** : * * **..*:.**.:***:******

BamA-Ec   SSTVRGFQSNTIGPKAVYFPHQASNY-DPDYDYECATQDGAKDLCKSDDAVGGNAMAVAS715
BamA-Kp   SSTVRGFQSNTIGPKAVYFPASSRHDDDDSYDNECKST--ESAPCKSDDAVGGNAMAVAS714
BamK-Kp   SSSVRGFSSNTIGPKAAYYRCNGSESS---Y--------SACPLDASSDAVGGNAMAVLN709
          **:****.********.*:  .. .     *              *.********** .

                                        L7
BamA-Ec   LEFITPTPFISDKYANSVRTSFFWDMGTVWDTNWDSSQ---YSGYPDYSDPSNIRMSAGI772
BamA-Kp   LELITPTPFISDKYANSVRTSVFWDMGTVWDTHWDSSA---YAGYPDYSDPSNIRMSAGI771
BamK-Kp   SEFIIPTPFVNDKYADSLRTSLFVDAGTVWSTSWHNTAQTLAAGIPDYGDPSHIRLSAGI769
          *:* ****:.****:*:***.* * ****.* *..:     :* ***.***:**:****

BamA-Ec   ALQWMSPLGPLVFSYAQPFKKYDGDKAEQFQFNIGKTW                      810
BamA-Kp   AVQWMSPLGPLVFSYAQPFKKYDGDKAEQFQFNIGKTW                      809
BamK-Kp   AVQWMSPLGPLVFSWAEPFKKYDGDKAEQFQFNIGKTW                      807
          *:************:*:*********************
```

B

```
-259  AGGCAAATCTAAATATAGTACTGGTGTTGGCCATTAGAACCATGCGCTTC

-200  AGAAAAAGTTCCATCATTAAATATATTTTTAAAGATGCTTTAACATCCAT

-150  TTTTTCTCCATTTTGTCGCCATAATTGCATGTAATTCTGCGCCTCGCTCA

-100  GGTTATTTTCTGTCGCATATTTTAGAGATGCATCCTGAACAAATAAGCTC

-50   AACGCCAGCAGGACACTGCTATTGCTCATTATCTCGTAGACGACACATTCATG
```

SI Fig. S3. (**A**) Amino acid sequence alignment for the β-barrel domains of *E. coli* BamA, *K. pneumoniae* BamA, and *K. pneumoniae* BamK. Positions where substitutions confer resistance to PTB1-1 are indicated with triangles. Black triangles identify positions only selected in *E. coli* and red triangles identify positions selected in both *E. coli* and *K. pneumoniae*. (**B**) DNA sequence of the *K. pneumoniae bamK* promoter region as described in (1). The -35 (TTCAGA, green), -10 (TATATT, green), ribosome binding site (AGACGA, blue), and the translation initiation codon (ATG, gold) are underlined and bold. A putative BaeS binding site matching the reported consensus binding motif (TTTTTCTCCATDATTGGC where D indicates G, A, or T) (2) in pink is underlined and bold.

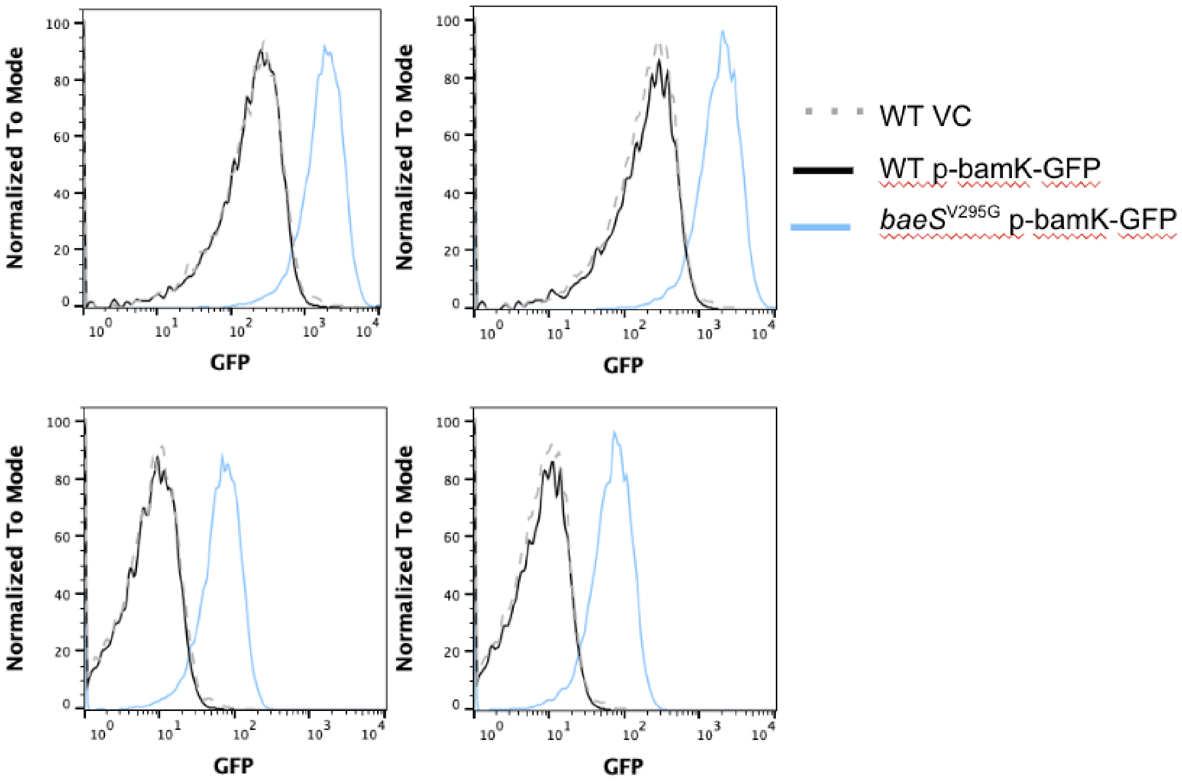

SI Fig. S4. Expression of *bamK-gfp* is induced in the PTB1-1-resistant *E. coli baeS*^{V295G} mutant. Expression of a P_{bamK}-GFP transcriptional reporter was measured by flow cytometry in *E. coli* wild-type (solid black line, WT p-bamK-GFP) and *baeS*^{V295G} mutant (solid blue line, *baeS*^{V295G} p-bamK-GFP) strains. A *E. coli* wild-type strain with an empty vector (WT VC) is shown as a dashed gray line. Two technical replicates of biological duplicates were run for each strain and all traces are shown.

SI Table S1. Frequencies of resistance (FOR) to BamA-targeting macrocycle PTB1-1 for *E. coli* and *K. pneumoniae* strains.

**Bacterial strains**	**Growth media**	**PTB1-1 MIC (µM)**	**FOR**
E. coli ATCC 25922	MHB	6.25	$1.4x10^{-8}$
K. pneumoniae ATCC 700721	MHB	12.5	$4.9x10^{-7}$
E. coli ATCC 25922	LB	0.78	$5.2x10^{-8}$
K. pneumoniae ATCC 700721	LB	3.12	$1.6x10^{-7}$

SI Table S2. Genes containing spontaneous, off-target (non-*bamA*) PTB1-1-resistance mutations selected in *K. pneumoniae*. Identifiers, parent strains, and specific mutants are noted in SI Table S3.

***K. pneumoniae* ancestry strain**	**isolates[1]**	***baeS* mutants**	***ramR* mutants**	***oqxR* mutants**
ATCC 700721	31[2]	18	3	7
ATCC 43816	20[3]	8	11	0

[1] total number of resistant clones subject to whole genome sequencing for each background; genes in which mutations were identified more than once are counted (*baeS*, *ramR*, and *oqxR*).
[2] For ATCC 700721, two spontaneous resistant isolates had no mutation and a single spontaneous resistant mutant had a mutation in *ccrB* (KPN_RS11140). See **SI Table S3** for additional details.
[3] For ATCC 43816, a single spontaneous resistant mutant was identified in *yadH*. See **SI Table S3** for additional details.

SI Table S3. Spontaneous, off-target PTB1-1-resistant mutations in *K. pneumoniae* identified by whole genome sequencing. Only variants present in at least 40% of mapped reads at the site of variation are reported and the frequency of each variant is indicated in parentheses.

Identifier	Background strain	Genomic mutations
GNE 5265	ATCC 700721	none identified
GNE 5266	ATCC 700721	BaeS [KPN_RS13610] V295G (100%)
GNE 5267	ATCC 700721	BaeS [KPN_RS13610] p151_163del (77%)
GNE 5268	ATCC 700721	none identified
GNE 5269	ATCC 700721	BaeS [KPN_RS13610] G446D (99%)
GNE 5270	ATCC 700721	BaeS [KPN_RS13610] V295G (100%)
GNE 5271	ATCC 700721	BaeS [KPN_RS13610] V295G (100%)
GNE 5272	ATCC 700721	BaeS [KPN_RS13610] G166D
GNE 5273	ATCC 700721	BaeS [KPN_RS13610] V295G (100%)
GNE 5274	ATCC 700721	BaeS [KPN_RS13610] G166D (100%), KPN_RS32170* (hypothetical protein) T32P (100%)
GNE 5275	ATCC 700721	BaeS [KPN_RS13610] V295G (100%)
GNE 5276	ATCC 700721	BaeS [KPN_RS13610] V295G (100%)
GNE 5277	ATCC 700721	BaeS [KPN_RS13610] V295G (100%)
GNE 5278	ATCC 700721	BaeS [KPN_RS13610] V295G (100%)
GNE 5279	ATCC 700721	CcrB [KPN_RS11140] P151M (100%)
GNE 5371	ATCC 700721	BaeS [KPN_RS13610] V295G (100%)
GNE 5372	ATCC 700721	Δ encompassing part of *mdtABCD-baeSR* operon [KPN_RS13590, KPN_RS13595, KPN_RS13600, KPN_RS13605, KPN_RS13610, KPN_RS13615]
GNE 5373	ATCC 700721	OqxR [KPN_RS15925] insertion
GNE 5374	ATCC 700721	BaeS [KPN_RS13610] Q188K (100%)
GNE 5376	ATCC 700721	Δ encompassing *oqxR* [KPN_RS15925]
GNE 5377	ATCC 700721	OqxR [KPN_RS15925] insertion
GNE 5378	ATCC 700721	OqxR [KPN_RS15925] insertion
GNE 5379	ATCC 700721	BaeS [KPN_RS13610] V295G (100%)
GNE 5380	ATCC 700721	BaeS [KPN_RS13610] V295G (100%)
GNE 5381	ATCC 700721	BaeS [KPN_RS13610] V295G (100%)
GNE 5382	ATCC 700721	BcsE [KPN_RS20985] Q199H (100%), RamR [KPN_RS03000] T119P (100%)

GNE 5383	ATCC 700721	BcsE [KPN_RS20985] Q199H (100%), RamR [KPN_RS03000] T119P(100%), g.1820662A>G
GNE 5384	ATCC 700721	BcsE [KPN_RS20985] Q199H (100%), RamR [KPN_RS03000] T119P (100%)
GNE 5385	ATCC 700721	OqxR [KPN_RS15925] insertion
GNE 5386	ATCC 700721	OqxR [KPN_RS15925] insertion
GNE 5387	ATCC 700721	OqxR [KPN_RS15925] insertion
GNE 6043	ATCC 43816	OqxR [VK055_RS23015] p11_20del (85%)
GNE 6044	ATCC 43816	OqxR [VK055_RS23015] I74S (100%)
GNE 6045	ATCC 43816	OqxR [VK055_RS23015] I74S (100%)
GNE 6046	ATCC 43816	RamR [VK055_RS09935] A17V (99%)
GNE 6047	ATCC 43816	RamR [VK055_RS09935] E175* (100%)
GNE 6048	ATCC 43816	OqxR [VK055_RS23015] p59ins66 (44%)
GNE 6049	ATCC 43816	RamR [VK055_RS09935] A72fs (98%)
GNE 6050	ATCC 43816	RamR [VK055_RS09935] A22T (100%)
GNE 6051	ATCC 43816	OqxR [VK055_RS23015] p59ins66 (46%)
GNE 6052	ATCC 43816	OqxR [VK055_RS23015] A19V (100%)
GNE 6053	ATCC 43816	OqxR [VK055_RS23015] M89P (100%)
GNE 6054	ATCC 43816	RamR [VK055_RS09935] L44fs (60%)
GNE 6055	ATCC 43816	RamR [VK055_RS09935] L44fs (62%)
GNE 6056	ATCC 43816 *ΔbamK*	RamR [VK055_RS09935] T119P (99%)
GNE 6057	ATCC 43816 *ΔbamK*	YadH [VK055_RS12190] P31M (99%)
GNE 6058	ATCC 43816 *ΔbamK*	Genomic deletion: OqxR [VK055_RS23015], GbuR [VK055_RS23010]
GNE 6059	ATCC 43816 *ΔbamK*	RamR [VK055_RS09935] Q23* (100%)
GNE 6060	ATCC 43816 *ΔbamK*	RamR [VK055_RS09935] V123fs (73%)
GNE 6061	ATCC 43816 *ΔbamK*	FabH [VK055_RS06920] A143E (100%) and g.2008677del (upstream of RamR [VK055_RS09935])
GNE 6062	ATCC 43816 *ΔbamK*	RamR [VK055_RS09935] A2D (100%)

***Formerly KPN_RS04200 in 15-JUN-2016 archival annotation of accession NC_009648.1**

SI Table S4. *K. pneumoniae* efflux mutants do not increase sensitivity to BamA-targeting macrocycle PTB1-1 in the *baeS*^{V295G}, *ramR*^{A72fs}, or *oqxR*^{V19A} mutant strain backgrounds. In all cases, an identical MIC was observed for two biological replicates.

***K. pneumoniae* strain**	**PTB1-1 MIC (μM)**
WT ATCC 43816	6.25
baeS^{V295G}	>100
baeS^{V295G} Δ*mdtAB*	>100
ramR^{A72fs}	50
ramR^{A72fs} Δ*acrAB*	50
oxqR^{V19A}	50
oqxR^{V19A} Δ*mexEF*	50

SI Table S5. Strains, plasmids, and primers used in this study.

Identifier	Background strain	Relevant genotype	Resistance	Source
GNE 178	*E. coli* ATCC 25922	WT		ATCC
GNE 115	*E. coli* BW25113	WT		(3)
GNE 132	*K. pneumoniae* ATCC 700721	WT		ATCC
GNE 649	*K. pneumoniae* ATCC 43816	WT		ATCC
GNE 130	*K. aerogenes* ATCC 13048	WT		ATCC
GNE 19	*E. cloacae* ATCC 222	WT		ATCC
GNE 131	*E. cloacae* ATCC 13047	WT		ATCC
GNE 230	*P. aeruginosa* PA14	WT		(4)
GNE 21	*A. baumannii* ATCC 19606	WT		ATCC
GNE 23	*S. aureus* USA300	WT		ATCC
GNE 6121	*B. subtilis RIK1285*	WT		Takara Bio
KMS 1715^{+}	*K. pneumoniae* ATCC 700721	$BamA^{G437C}$	PTB-1^{R}	This study
KMS 1716^{+}	*K. pneumoniae* ATCC 700721	$BamA^{G437S}$	PTB-1^{R}	This study
KMS 1010^{+}	*K. pneumoniae* ATCC 43816 Δ*bamK*	$BamA^{D464H}$	PTB-1^{R}, Kan^{R}	This study
KMS 1717^{+}	*K. pneumoniae* ATCC 700721	$BamA^{D500A}$	PTB-1^{R}	This study
KMS 999^{+}	*K. pneumoniae* ATCC 43816	$BamA^{D500V}$	PTB-1^{R}	This study
GNE 143	*E. coli* MG1655 conditional *bamA*	Δ*bamA*::P_{BAD}-*bamA*	$Carb^{R}$, Kan^{R}	(5)
GNE 1101	*E. coli* MG1655 conditional *bamA* *waaD* mutant	Δ*waaD* Δ*bamA*::P_{BAD}-*bamA*	$Carb^{R}$, Kan^{R}, Cm^{R}	(5)
GNE 6209	*E. coli* O157 ATCC 43895	WT 933W phage		ATCC
KMS 1429	*E. coli* O157 ATCC 43895	Δ*stx2*	Cm^{R}	This study

GNE 5406	*K. pneumoniae* ATCC 43816	Δ*bamK*	Kan^R	This study
GNE 6085⁺	*K. pneumoniae* ATCC 43816	*baeS*^{V295G}	PTB-1^R	This study
GNE 5986	*K. pneumoniae* ATCC 43816 *baeS*^{V295G}	Δ*bamA baeS*^{V295G}	PTB-1^R, Kan^R	This study
GNE 5987	*K. pneumoniae* ATCC 43816 *baeS*^{V295G}	Δ*bamK baeS*^{V295G}	PTB-1^R, Kan^R	This study
GNE 5272⁺	*K. pneumoniae* ATCC 700721 *baeS*^{G166D}	*baeS*^{G166D}	PTB-1^R	This study
GNE 5374⁺	*K. pneumoniae* ATCC 700721 *baeS*^{Q188K}	*baeS*^{Q188K}	PTB-1^R	This study
GNE4269⁺	*K. pneumoniae* ATCC 700721 *baeS*^{G446D}	*baeS*^{G446D}	PTB-1^R	This study
GNE 6049⁺	*K. pneumoniae* ATCC 43816 *ramR*^{A72fs}	*ramR*^{fs}	PTB-1^R	This study
GNE 6052⁺	*K. pneumoniae* ATCC 43816 *oqxR*^{A19V}	*oqxR*^{A19V}	PTB-1^R	This study
KMS 1352	*K. pneumoniae* ATCC 43816 Δ*mdtAB baeS*^{V295G}	Δ*mdtAB baeS*^{V295G}	PTB-1^R, Kan^R	This study
KMS 1358	*K. pneumoniae* ATCC 43816 Δ*acrAB ramR*^{A72fs}	Δ*acrAB ramR*^{fs}	PTB-1^R, Kan^R	This study
KMS 1353	*K. pneumoniae* ATCC 43816 Δ*oqxAB oqxR*^{A19V}	Δ*oqxAB oqxR*^{V19A}	PTB-1^R, Kan^R	This study

⁺ Spontaneous mutants obtained by selection noted by plus (+)
Antibiotic resistance: Kan^R (Kanamycin), $Carb^R$ (Carbenicillin), Cm^R (Chloramphenicol)

Plasmid	Description	Antibiotic resistance	Source
pBla-Short	Empty vector	$Carb^R$	(5)
p-*bamA*-Ec	pBla-short constitutively expressing *bamA*^{Ec}	$Carb^R$	(5)
p-*bamA*-Kp	pBla-short constitutively expressing *bamA*^{Kp}	$Carb^R$	This study
p-*bamK*-Kp	pBla-short constitutively expressing *bamK*^{Kp}	$Carb^R$	This study
p-*bamK*-GFP	pBla-short DASHER GFP with *bamK* promoter (216 bp upstream of ATG)	$Carb^R$	This study

Primer	Description	Sequences
pBla_*bamA*Ec GA F	PCR for $P_{bamA\text{-}Ec}$	CGTTATTATGCGTTCTTCCTAACTAACTC
pBla_*bamA*Ec GA R		GAGTAAGCTTAACATAAGGAGGAAAA
*bamK*_Kp GA F	construct $P_{bamK\text{-}Kp}$	gaagaacgcataataacgATGTTAAAAAAGACTCATATCATCAGC
*bamK*_Kp GA R		cttatgttaagcttactcTTACCAGGTTTTGCCGATATTAAAC
*bamA*_Kp GA F	construct $P_{bamA\text{-}Kp}$	gaagaacgcataataacgATGGCGATGAAAAAGTTG
*bamA*_Kp GA F		cttatgttaagcttactcTTACCAGGTTTTACCAATGTTAAAC
bamK Kp KO F	construct *K. pneumoniae* Δ*bamK*	CAACGCCAGCAGGACACTGCTATTGCTCATTATCTCGTAGACGAC<br>ACATTCgtgtaggctggagctgcttc
bamK Kp KO R		GAGGACAGTCCACAGAATATAGCCACGCCGGTCTATCAGGCCCC<br>GTTGGAGCCcatatgaatatcctccttagttcctattc
bamK Kp outside F	Confirmation primers *K. pneumoniae* Δ*bamK*	CATTCCTAACGAGATCCTTC
bamK Kp outside R		CATTCCTTAGCGATAATATCGAC
bamA Kp KO F	construct *K. pneumoniae* Δ*bamA*	CAATGATTTCTCTCGGTTATAAGAGAGTTTGTTAGGAAGAACGCAT<br>AATAACGgtgtaggctggagctgcttc
bamA Kp KO R		CGTTATCGCCTACAGTCAGCGCTATACTGCACTTGCATTCCGTTG<br>CCGCCAACAAcatatgaatatcctccttagttcctattc
bamA Kp outside F	confirmation primers *K. pneumoniae* Δ*bamA*	GGTTGATTTATTATCTGATGTTCC
bamA Kp outside R		CCACTTTTTCACAATAAACTCC
stx2 Ec KO F	construct *E. coli* Δ*stx2*	caactgtcaactgactgaattgtgacacagattacacttgttacccacataccacgaatcaggtt<br>atgccCGGAATAGGAACTTCAAGATCC
stx2 Ec KO R		gcttttgcgggcctttttatatctgcgccgggtctggtgctgattacttcagccaaaaggaacacct<br>gtatcttcagagcgcttttgaagc
stx2 Ec outside F	Confirmation primers *E. coli* Δ*stx2*	GACACCTTATACACCAGTCG
stx2 Ec outside R		CCATATCACATACCGCCATTAG
bamK promoter-DASHER GA R	construct P_{bla}-*bamK*-GFP	ttgtcgacggagctcCTGATACGTGTCCAGATCAAC
bamK promoter-DASHER GA F		ccggcgtagaggatcCCTTCCCCCTTTCCATTAATAAG
mdtAB Kp KO F	construct *K. pneumoniae* Δ*mdtAB*	C*G*G*C*CCCTTCCCTCACTAAACTAGTATCATTCAGCGAAACGCT<br>TCAGGATGAGACCGTAAACTAgtgtaggctggagctgcttc
mdtAB Kp KO R		G*T*G*A*TGGCCAGCGAGATGAGGATCGTCGCCACCGGGCGGTA<br>AATGAAGAGGGCGAAAAACTTCACcatatgaatatcctccttagttcctattc

mdtAB Kp outside F	Confirmation primers _K. pneumoniae_ Δ_mdtAB_	GATATCACCCCGGATATCGG
mdtAB Kp outside R		GAGGAGGTCATCTCATTGACC
acrAB Kp KO F	construct _K. pneumoniae_ Δ_acrAB_	G*T*G*G*GTTTGTGGTTGTTTGAGCCACTGAACATTTTGAAATTGG ACACTCGAGGTTTACATgtgtaggctggagctgcttc
acrAB Kp KO R		G*T*G*C*CTTGCCAGCCAGTGATAAAAAAGGGCCGCGGTAGCGG CCCTTTGTTCAGGAGTGAAGAcatatgaatatcctccttagttcctattc
acrAB Kp outside F	Confirmation primers _K. pneumoniae_ Δ_acrAB_	GTTTCACGTGCCTGTTGTTTG
acrAB Kp outside R		CCACAGCCGGAGAAATAGAG
oqxAB Kp KO F	construct _K. pneumoniae_ Δ_oqxAB_	G*A*C*A*AAGGAAGTGGCGCGGCTTCTCACGCTGCGTCTTGCCC GGCGGCTACATTTACCGGAATAAAAATAgtgtaggctggagctgcttc
oqxAB Kp KO R		C*A*T*T*TTCTGGTGACGAAAAAAAAACCGCCTTCAAATTGTGAAGG CGGTTTTTTTGTTATCTGCTGCAGGCcatatgaatatcctccttagttcctattc
oqxAB Kp outside F	Confirmation primers _K. pneumoniae_ Δ_oqxAB_	GTGCAACAATTATTCTTGCAC
oqxAB Kp outside R		GGCTTCGTTAAACGTCCTC
bamA-Kp F	qRT-PCR	cccgacgccgtttattagt
bamA-Kp R		ttttgaacggttgggcgtag
bamK-Kp F	qRT-PCR	gtcgtgaaatgcgccaaatg
bamK-Kp F		ccaggcccacattgaatgag
rpoD-Kp F	qRT-PCR	caatgaccatctgccggaag
rpoD-Kp R		cttcggcagcatcttcatcc
Kp bamA -250bp upstream F	_K. pneumoniae bamA_ primers for Sanger sequence analysis	GGTTGATTTATTATCTGATGTTCC
Kp bamA -100bp downstream R		CCACTTTTTCACAATAAACTCC
Kp bamA 200bp F		GACGATGATATCAGTAACAC
Kp bamA 725bp F		CGTTTCAACATCGATTCTAC
Kp bamA 1300bp F		GCTTCAACTTCGGTATCG
Kp bamA 1900bp F		GATCATCAGTGGGTAGTAC

*** phosphorothioate bond positions are noted by asterisks (*)**

Supplemental references

1. Torres VVL, Heinz E, Stubenrauch CJ, Wilksch JJ, Cao H, Yang J, Clements A, Dunstan RA, Alcock F, Webb CT, Dougan G, Strugnell RA, Hay ID, Lithgow T. 2018. An investigation into the Omp85 protein BamK in hypervirulent *Klebsiella pneumoniae*, and its role in outer membrane biogenesis. Molecular Microbiology 109:584–599.

2. Nishino K, Honda T, Yamaguchi A. 2005. Genome-Wide Analyses of *Escherichia coli* Gene Expression Responsive to the BaeSR Two-Component Regulatory System. J Bacteriol 187:1763–1772.

3. Datsenko KA, Wanner BL. 2000. One-step inactivation of chromosomal genes in *Escherichia coli* K-12 using PCR products. Proceedings of the National Academy of Sciences 97:6640–6645.

4. Rahme LG, Stevens EJ, Wolfort SF, Shao J, Tompkins RG, Ausubel FM. 1995. Common Virulence Factors for Bacterial Pathogenicity in Plants and Animals. Science 268:1899–1902.

5. Storek KM, Auerbach MR, Shi H, Garcia NK, Sun D, Nickerson NN, Vij R, Lin Z, Chiang N, Schneider K, Wecksler AT, Skippington E, Nakamura G, Seshasayee D, Koerber JT, Payandeh J, Smith PA, Rutherford ST. 2018. Monoclonal antibody targeting the β-barrel assembly machine of *Escherichia coli* is bactericidal. Proceedings of the National Academy of Sciences of the United States of America 115:3692–3697.
